# 

**Published:** 1880-05

**Authors:** 


### CONTENTS.

COMMUNICATIONS.

Address at the Commencement of the Dental College, at Ann Arbor, March 24th, 1880 ........ 201
Afternoon Session, March 3rd, 1880; Continued from Page 185........ 218

GRADUATES IN DENTISTRY, 1880.

Baltimore College of Dental Surgery ........ 233
Ohio College of Dental Surgery........ 234
Pennsylvania College of Dental Surgery........ 235
University of Tennessee—Dental Department........ 237
Philadelphia Dental College........ 237
New York College of Dentistry........ 238
Dental Department of Vanderbilt University........ 239
Dental Department of the University of Michigan........ 239
Indiana Dental College........ 240

SCIENTIFIC NEWS ........ 241

Miscellaneous Notes ........ 242

EDITORIAL.

Indiana State Dental Society........ 244

